# Patients’ and rheumatologists’ perceptions on preventive intervention in rheumatoid arthritis and axial spondyloarthritis

**DOI:** 10.1186/s13075-020-02314-9

**Published:** 2020-09-15

**Authors:** Laurette van Boheemen, Janne W. Bolt, Marieke M. ter Wee, Henriëtte M. de Jong, Marleen G. van de Sande, Dirkjan van Schaardenburg

**Affiliations:** 1 Department of Rheumatology, Amsterdam Rheumatology & Immunology Center (ARC)—Reade, PO box 58271, 1040 HG Amsterdam, the Netherlands; 2 Department of Rheumatology and Clinical Immunology, Amsterdam Rheumatology & Immunology Center (ARC), Amsterdam UMC, University of Amsterdam, Amsterdam, the Netherlands; 3 Department of Rheumatology, Amsterdam Rheumatology & Immunology Center (ARC), Amsterdam UMC, VUmc, Amsterdam, the Netherlands; 4 Department of Epidemiology and Biostatistics, Amsterdam UMC, VUmc, Amsterdam, the Netherlands

**Keywords:** Rheumatoid arthritis, Axial spondyloarthritis, Primary prevention, Lifestyle intervention, Preventive medication

## Abstract

**Background:**

Individuals at risk of developing rheumatoid arthritis (RA) may benefit from lifestyle or pharmacological interventions aimed at primary prevention. The same may apply to individuals at risk of axial spondyloarthritis (axSpA). Our aim was to investigate and compare the willingness of individuals at risk of RA or axSpA and rheumatologists to initiate preventive intervention.

**Methods:**

Individuals at risk of RA (arthralgia and anti-citrullinated protein antibodies and/or rheumatoid factor positivity without arthritis (RA-risk cohort; *n* = 100)), axSpA (first-degree relatives of HLA-B27-positive axSpA patients (SpA-risk cohort; *n* = 38)), and Dutch rheumatologists (*n* = 49) completed a survey on preventive intervention which included questions about disease perception, lifestyle intervention, and preventive medication.

**Results:**

At-risk individuals reported willingness to change median 7 of 13 lifestyle components in the areas of smoking, diet, and exercise. In contrast, 35% of rheumatologists gave lifestyle advice to ≥ 50% of at-risk patients. The willingness to use 100% effective preventive medication without side effects was 53% (RA-risk), 55% (SpA-risk), and 74% (rheumatologists) at 30% disease risk which increased to 69% (RA-risk) and 92% (SpA-risk and rheumatologists) at 70% risk. With minor side effects, willingness was 26%, 29%, and 31% (at 30% risk) versus 40%, 66%, and 76% (at 70% risk), respectively.

**Conclusions:**

Risk perception and willingness to start preventive intervention were largely similar between individuals at risk of RA and axSpA. Although the willingness to change lifestyle is high among at-risk individuals, most rheumatologists do not advise them to change their lifestyle. In contrast, rheumatologists are more willing than at-risk patients to start preventive medication.

## Background

The recognition of a preclinical phase in rheumatoid arthritis (RA) has opened up possibilities to investigate preventive treatment strategies [1]. In the phase before clinical arthritis onset, characteristic symptoms and biomarkers are often already present [2]. This has enabled the development of prediction models aiming to identify individuals with a high risk of developing RA who qualify for preventive intervention [3, 4]. Environmental factors including lifestyle are important in RA development and lifestyle intervention may delay or prevent RA onset [5–7]. In addition, several placebo controlled clinical trials in individuals at increased risk of RA have been performed or are ongoing [8–10].

As prediction and prevention of RA evolve, it is increasingly likely that at-risk individuals and health care professionals are faced with decisions about whether to initiate preventive treatment. Patients’ perceptions of risk and benefit have an important influence on their willingness to start treatment and studies in the fields of oncology and cardiovascular disease have shown that the willingness is the main determining factor of the effectiveness of a preventive approach [11, 12]. However, information about perceptions of individuals at risk of RA regarding preventive treatment is still limited. Previous studies in first-degree relatives (FDRs) of RA patients showed that many were willing to make lifestyle changes such as weight loss or diet changes and that their willingness to start preventive medication was primarily influenced by perceived risk of RA development, medication effectiveness, and potential side effects [13–15]. Furthermore, the opinion of the health care professional might be an important attribute involved in the patient’s decision whether or not to take preventive treatment [16, 17]. However, FDRs are mainly asymptomatic individuals and perceptions in this group may differ from symptomatic at risk individuals [18]. It is important to better understand perceptions of symptomatic at risk individuals since they are the target population for current ongoing preventive intervention trials.

In other inflammatory rheumatic diseases, such as axial spondyloarthritis (axSpA), even less is known about the effectiveness of very early treatment. However, it is plausible that a preclinical stage also exists in which treatment may delay or prevent disease onset [19]. First-degree relatives of axSpA patients are at increased risk of developing this disease and a substantial part also experience symptoms such as chronic back pain [20]. The willingness of individuals at risk of axSpA to start preventive medication has recently been studied and was overall high in case of a clearly increased risk and no medication side effects [21]. Comparing willingness to start preventive treatment between individuals at risk of RA and individuals at risk of axSpA can provide information on the generalizability of the findings. At risk individual’s perceptions should be taken into account when designing preventive trials and, when an intervention has proved effective, will be important in optimizing acceptance and adherence to preventive treatment.

The objectives of this study were (1) to investigate the willingness of symptomatic individuals at increased risk of developing RA, and individuals at increased risk of axSpA to initiate preventive treatment, (2) to evaluate which factors influence at risk individuals’ willingness to initiate preventive treatment, and (3) to compare willingness between individuals at risk of RA, individuals at risk of axSpA, and rheumatologists.

## Methods

### Study population

All participants included in the Reade seropositive arthralgia cohort (RA-risk) [22] between February 2010 and June 2019 were asked to complete a survey about their perceptions on preventive intervention. This cohort includes individuals at increased risk of RA defined by having arthralgia and testing positive for at least one serologic marker: anti-citrullinated protein antibodies (ACPA; > 10 kU/l) and/or rheumatoid factor (RF; > 5 kU/l) with no history of clinically diagnosed arthritis. In addition, all individuals included in the Academic Medical Center Amsterdam (AMC) Pre-SpA cohort, a spondyloarthritis risk cohort (further referred to as SpA-risk) [20], between October 2018 and February 2020 were asked to complete the same survey. A shorter version of the current survey was previously sent to participants from the SpA-risk cohort included before October 2018 [21]. The current survey was sent only to participants that were included after the previous survey study ended, to prevent data collection in the same participants. The SpA-risk cohort includes healthy FDRs between 18 and 40 years old of HLA-B27-positive axSpA patients, part of whom are symptomatic and/or HLA-B27 positive. Participants were not diagnosed with axSpA at the time of the baseline visit and had no previously confirmed non-rheumatic diagnosis of backpain. Also, the members of the Dutch Society for Rheumatology (rheumatologists and rheumatology residents) were approached by a notice in the monthly digital newsletter to complete a survey. Patients gave their written informed consent and health care professionals gave their consent by completing the survey. The ethics board of the Slotervaart Hospital and Reade (RA-risk and health care professionals) and the ethics board of the AMC (Pre-SpA cohort) approved the study protocol.

### Survey

Participants from the RA-risk and SpA-risk cohort received the same survey, comprising 12 statements about perception of disease, disease risk, and ethical views on participation in an at risk cohort, which were scored on a visual analog scale (VAS) from 0 (totally disagree) to 10 (totally agree). Thirteen questions about current lifestyle (including smoking, alcohol consumption, exercise, and diet) and willingness to change lifestyle factors were scored on a VAS from 0 (not willing) to 10 (very willing), concluding with a question on the total number of listed lifestyle changes participants were willing to make (0–13). Six preventive medication scenarios were presented, adjusted for either RA or axSpA, that differed in disease risk, drug treatment effectiveness, and potential side effects (Additional files 1 and 2). For each scenario, participants could answer to what degree they would initiate preventive treatment on a 5-point Likert scale. In a multiple choice question, participants could indicate which aspects were most important when considering preventive medication use. These questions were based on the survey previously used in a different subset of participants from the SpA-risk cohort [21]. Health care professionals received an adjusted version of the RA-risk survey about starting preventive intervention in patients at risk of RA (Additional file 3). It contained 3 multiple choice questions about lifestyle advice in their current clinical practice together with the six preventive medication scenarios, followed by the multiple choice question to indicate which aspects were most important when considering preventive medication and a question about minimum risk level for considering preventive treatment (ranging from 10 to 100%).

### Statistical analyses

Disease perceptions (VAS), ethical aspects of cohort participation (VAS), current lifestyle habits (presence or absence), willingness to change lifestyle (VAS), and willingness to start preventive medication (categories) were explored using descriptive statistics and compared between individuals at risk of RA and individuals at risk of axSpA using *t* test (for normally distributed continues data) or Mann-Whitney *U* test (for non-normally distributed continuous data) and Chi-square test or Fisher’s exact test (binary data). Additionally, rheumatologists’ willingness to prescribe medication (categories) and to offer lifestyle advice (categories) were compared with RA-risk individuals’ views by applying the same statistical tests as mentioned above.

Associations between disease perception, clinical features (age, sex, pain), and willingness to change lifestyle, were tested using linear regression analysis. The total number of lifestyle changes participants were willing to make was entered as the dependent variable and the disease perception and clinical features were entered as independent variables. To test whether disease perception and clinical features affected willingness to start preventive medication, a generalized estimating equations (GEE) model was used. This corrects for the fact that each individual’s answers to each scenario were related to their answers in previous scenarios. Treatment willingness was dichotomized into willing (“Yes” and “I probably would”) and unwilling (“I don’t know,” “I would probably not,” and “No”). Individuals’ answers were entered as the dependent variable, and the disease perception and clinical features were entered as independent variables.

## Results

In total, 133 individuals at risk of RA and 52 individuals at risk of axSpA were asked to complete the survey. The Dutch Society for Rheumatology digital newsletter was sent to 439 members. Response rates were 75% (*n* = 100), 73% (*n* = 38), and 11% (*n* = 49), respectively. RA-risk responders were slightly older than non-responders (mean age 54 vs 49, *p* = 0.042) but did not differ regarding sex, autoantibody status, or level of RA risk. SpA-risk responders did not differ from non-responders regarding age, sex, backpain, or HLA-B27 status. Cohort characteristics are shown in Table 1. As expected, the mean age of RA-risk individuals was higher than of axSpA-risk individuals. Data on disease risk perception and ethical aspects regarding cohort participation are shown in Table 2. Overall, participants considered RA and axSpA to be a serious disease (median VAS 6.5 (IQR 5–8; RA-risk) and 6 (IQR 4–8; SpA-risk)). Despite some concern about their increased risk (median VAS 5 (IQR 2–6) and 3 (IQR 1–5), respectively), most participants did not expect to develop the disease (median VAS 3 (IQR 1–5) for both). Nevertheless, they did not mind to be extra confronted with the risk of developing a disease as a result of study participation (median VAS 1 (IQR 0–2) and 1 (IQR 0–1), respectively). The most important motives to participate in the at-risk cohorts were the wish to contribute to science and to have their symptoms monitored closely.

**Table 1:** Baseline characteristics of the RA-risk cohort and SpA-risk cohort

	RA-risk cohort, *n* = 100	SpA-risk cohort, *n* = 38
Age, mean (SD)	54 (11)	28 (7)
Female sex, *n* (%)	71 (71)	25 (66)
Current smoker, *n* (%)	17 (17)	8 (21)
VAS joint pain, median (IQR)	24 (5–50)	–
Self-reported history of swollen joints (median, IQR)	0 (0–0)	–
RF positive, *n* (%)	72 (72)	–
ACPA positive, *n* (%)	37 (37)	–
High RA-risk (≥ 38% in 4 years)*, *n* (%)	40	-
Back pain present, *n* (%)	–	28 (74)
Inflammatory back pain present, *n* (%)	–	6 (21)
VAS back pain, median (IQR)	-	14 (0–36)
HLA-B27 positive, *n* (%)	–	20 (56)

*ACPA* anti-citrullinated protein antibodies, *RF* rheumatoid factor, *IQR* interquartile range, *RA* rheumatoid arthritis, *SD* standard deviation, *SpA* spondyloarthritis, *VAS* visual analog scale

*Retrospectively calculated using the clinical prediction score of van de Stadt et al. [23]

**Table 2:** Disease risk perception and ethical aspects regarding cohort participation based on visual analog scale

	RA-risk cohort	SpA-risk cohort
Q8 The thought of developing RA/SpA preoccupies me	4 (2–6)	3 (1–5)
Q9 I am certain that I will develop RA/SpA	3 (1–5)	3 (1–5)
Q10 RA/SpA is a severe disease	6.5 (5–8)	6 (4–8)
Q11 I am worried that I have an increased risk of developing RA/SpA	5 (2–6)	3 (1–5)
Q12 By participating in this research, I feel that I am extra confronted with the fact that I have an increased risk to develop RA/SpA	2 (1–5)	1.5 (1–4)
Q13 How objectionable is it for you to be extra confronted with the risk to develop RA/SpA by participation in this cohort?	1 (0–2)	1 (0–1)
Q14 By participating in this research there is more attention for my complaints	6 (2–8)	4.5 (1–5.25)
Q15 I think that by participating in this study I will receive earlier and better medical care upon RA/SpA development than if I did not participate in this study	7 (5–8)	7.5 (6–9)

Numbers are median (IQR)

*VAS* visual analog scale from 0 (totally disagree) to 10 (totally agree), *RA* rheumatoid arthritis, *SpA* spondyloarthritis

To decrease personal disease risk, all at risk individuals were willing to change at least 1 lifestyle component, with a median of 7 (IQR 4–10 (RA-risk) and 5–8 (SpA-risk)) out of 13 components in multiple areas. Overall, they were most willing to increase their fruit and vegetable intake according to the national guidelines for a healthy diet and to stop drinking sodas and fruit juices. In contrast, they were least willing to stop consuming dairy products and meat. There were no statistically significant differences in the reported daily intake of these products: intake of ≥ 2 servings of fruit, 55% (RA-risk) and 42% (SpA-risk); ≥ 250 g of vegetables, 71% (RA-risk) and 61% (SpA-risk); soda or fruit juices, 45% (RA-risk) and 68% (SpA-risk); dairy products, 98% (RA-risk) and 100% (SpA-risk); and meat, 95% (both). Ninety-five percent of smokers indicated that they would quit (RA-risk, 94%; SpA-risk, 100%); however, they scored their motivation to do so a 7 (IQR 6–8, RA-risk) and 6 (IQR 4.5–8, SpA-risk) out of 10. The willingness to increase daily physical exercise was higher among individuals at risk of axSpA (reported compliance with the national physical activity guideline 74% (RA-risk) and 58% (SpA-risk), *p* = 0.06) and the willingness to limit alcohol intake was higher among individuals at risk of RA (reported alcohol use 61% (RA-risk) and 79% (SpA-risk), *p* = 0.06). No other statistically significant differences in willingness to change lifestyle components were observed between the two cohorts. Only among individuals at risk of axSpA was preoccupation with the thought of developing the disease associated with a higher willingness to make lifestyle changes (beta 0.45, 95% CI 0.14–0.77).

Twenty-five percent of rheumatologists advised lifestyle changes to < 10% of their RA-risk patients and 35% advised lifestyle changes to ≥ 50% of RA-risk patients. The most frequently given advice was to stop smoking (by 96% in case of any lifestyle advice given) and to increase physical activity, mostly to facilitate weight loss (74%). Rheumatologists who offered little lifestyle advice indicated that this was mainly due to lack of time or lack of uniform evidence on the effect of lifestyle changes on decreasing RA risk. They did, however, believe it was part of their job and they had enough expert knowledge to give lifestyle advice.

Willingness to use or prescribe preventive medication in individuals at risk of RA, axSpA, and rheumatologists, based on 6 different scenarios, is shown in Table 3. Overall, rheumatologists were more willing than individuals at risk of RA to start preventive medication, except for the scenario in which medication would not prevent, but delay RA onset with 10 years. The willingness of individuals at risk of RA and axSpA was similar in the case of a 30% disease risk. At a 70% disease risk, individuals at risk of axSpA were more willing to use 100% effective medication. In the RA-risk cohort, men (GEE analysis: OR 2.88, 95% CI 1.37–6.05) and those who considered RA to be a serious disease were more willing to start preventive medication (GEE analysis: OR 1.17, 95% CI 1.04–1.32, Table 4). In the SpA-risk cohort, being older was associated with increased medication willingness (GEE analysis: OR per year 1.07, 95% CI 1.00–1.14), while disease perception showed no association. Overall, a decrease in disease risk from 70 to 30% and the expected occurrence of side effects significantly lowered treatment willingness (*p* < 0.05) in all groups (data not shown). For considering preventive medication, the most important aspects were the expected occurrence of side effects (RA-risk, 29%; SpA-risk, 31%; rheumatologists, 10%), the certainty that the medication would prevent the disease (RA-risk, 29%; SpA-risk, 31%; rheumatologists, 45%) and the likelihood that the disease would develop without medication (RA-risk, 29%; SpA-risk, 21%; rheumatologists, 43%). Of the rheumatologists, 27% indicated that a risk between 10 and 30% of developing RA within 3 years was sufficient to start preventive therapy, whereas another 33% of the rheumatologists preferred a 70% or higher risk before starting medication. No association was found between age (mean 46 years, SD 10) and sex (67% female) of rheumatologists and willingness to prescribe preventive treatment.

**Table 3:** Willingness to start preventive medication

Disease risk	At risk of RA (%)	At risk of axSpA (%)	Rheumatologists (%)	At risk of RA versus at risk of axSpA	At risk of RA versus rheumatologists
	100% effective medication, no side effects				
30%	53	55	74	*p* = 0.812	***p*** **= 0.017**
70%	69	92	92	***p*** **= 0.005**	***p*** **= 0.002**
	100% effective medication, minor side effects of immune suppression				
30%	26	29	31	*p* = 0.727	*p* = 0.554
70%	40	66	76	***p*** **= 0.007**	***p*** **< 0.001**
	100% effective medication, minor general side effects				
30%	40	47	88	*p* = 0.433	***p*** **< 0.001**
	Medication postpones disease development for 10 years, no side effects				
70%	61	66	57	*p* = 0.604	*p* = 0.652

*axSpA* axial spondyloarthritis, *RA* rheumatoid arthritis

**Table 4:** Association between clinical features and willingness to take preventive medication

	At risk of RA	At risk of axSpA
	OR (95% CI)	OR (95% CI)
Male sex OR (95% CI)	**2.88 (1.37–6.05)**	1.14 (0.38–3.41)
Age per year OR (95% CI)	0.99 (0.96–6.05)	**1.07 (1.00–1.14)**
VAS pain per point OR (95% CI)	1.00 (0.99–1.02)	0.99 (0.97–1.01)

*axSpA* axial spondyloarthritis, *CI* confidence interval, *OR* odds ratio, *RA* rheumatoid arthritis

## Discussion

Individuals at risk of RA or axSpA state that they are highly willing to make lifestyle changes, while most rheumatologists do not advise at-risk patients to do so, mostly due to a current lack of evidence. In contrast, rheumatologists are more willing to prescribe preventive medication than at-risk individuals are to use it. The willingness to use preventive medication of individuals at risk of RA or axSpA is similar at a lower disease risk (30%) while at a high risk (70%), individuals at risk of axSpA are more willing to use medication. Overall, willingness is higher in men (RA-risk), older persons (SpA-risk), those who consider the disease to be serious (RA-risk), and in persons who are preoccupied with developing the disease (SpA-risk). A decrease in disease risk and the expected occurrence of side effects significantly decreases willingness to use medication. Disease risk perception, ethical views on cohort participation, and willingness to change lifestyle to decrease disease risk are similar between individuals at risk of RA or axSpA.

The previously reported high willingness of FDRs of RA patients to change their lifestyle [13–15] is also observed in the present at-risk populations. The willingness to use preventive medication seems higher in symptomatic RA-risk individuals compared to asymptomatic FDRs. Finckh et al. reported that 38% of FDRs would be willing to use preventive medication at a 40% disease risk [13]. Compared to a 53% willingness of symptomatic at risk individuals at a 30% disease risk, this supports the rationale from qualitative studies that the presence of symptoms would increase willingness to use preventive medication [18]. The willingness to start preventive medication in the current group of SpA-risk individuals is comparable to the group that was included in the study of de Winter et al. [21], validating these results.

In case of a clearly increased disease risk, individuals at risk of axSpA may be more willing to use medication than individuals at risk of RA. This might be explained by the fact that the SpA-risk population is significantly younger and axSpA starts at a younger age, making the dilemma between disease risk without intervention and possible overtreatment more urgent. However, qualitative research is needed to determine the underlying motives. In both groups, potential side effects played an important role in the decision to start preventive medication, which confirms previous research reporting a large effect of expected mild side effects on decision-making, even if these would cease after stopping the medication [15]. Conversely, worries about disease development and severity increased preventive intervention willingness in both groups. VAS pain showed no association with intervention willingness, which might be explained by the fact that in individuals at risk of RA, symptoms usually fluctuate [24] and in the SpA-risk cohort, the overall VAS pain was low (median 14, IQR 0–36). It is important to note that, in contrast to the scenarios presented in the questionnaire, in reality, the risk of disease development is lower in SpA-risk individuals than in RA-risk individuals and this will affect individuals’ willingness to initiate preventive intervention in clinical practice [25].

A minority of rheumatologists sometimes advises individuals at risk of RA to stop smoking and lose weight, but the majority requires more evidence as to whether lifestyle changes reduce the risk of developing RA before implementing lifestyle advice into daily practice. Indeed, while some environmental risk factors have been identified, it has not yet been fully clarified how most of these influence autoimmunity [26] and how changing these factors affect RA-risk. To address this, we are currently performing a randomized controlled trial investigating the effects of a lifestyle intervention program on disease risk in individuals with (an increased risk of) RA. Furthermore, rheumatologists were more willing to prescribe preventive medication than individuals at risk of RA were willing to use medication. This is an important finding since the manner in which benefits and risks of treatment are presented to at-risk individuals influences health decisions and a positive attitude of the rheumatologist may encourage at risk individuals to decide for intervention [17]. However, minor side effects did not affect the rheumatologists’ decision to start medication, while it is a significant concern for at risk individuals. Therefore, despite their low levels of concern, rheumatologists should address at-risk persons’ worries about side effects and provide balanced education on potential side effects of preventive therapy in relation to personal disease risk.

A strength of this study is that it is the first study on preventive intervention willingness in a large group of symptomatic individuals at risk of RA. Furthermore, a comparison could be made with individuals at risk of axSpA and rheumatologists.

A limitation of our study is the possible channeling bias. Ethical views on cohort participation could be answered differently compared to those who chose to not participate in the study cohort. Also, the people who completed the questionnaire might be more inclined to start preventive treatment because they are more interested in the subject than those who did not complete the questionnaire; however, the overall response rate of at-risk individuals was high. Conversely, the response rate of rheumatologists was low, and it is uncertain whether these results are representative for Dutch rheumatologists. Additionally, the relatively low number of SpA-risk individuals compared to RA-risk individuals is considered a limitation of the current study. Furthermore, to create a clear and still practical survey, a limited number of questions were chosen per subject. Nevertheless, these give a good impression of ethical views regarding cohort participation, the overall willingness to start preventive intervention and important decision-making factors in at-risk individuals.

In summary, these results support the need for studies on the effect of lifestyle changes on disease risk. In addition, to facilitate future prevention trials using medication, we suggest research into optimal education of at-risk individuals about interpreting potential side effects of medication in relation to personal disease risk. Future trials should aim to include individuals who were recently informed about their personal risk, calculated using the currently available prediction tools, thereby closely resembling clinical practice.

## Conclusions

In conclusion, symptomatic individuals at risk of RA seem very willing to make lifestyle changes and the majority is willing to use preventive medication, especially in case of a clearly increased risk or the perception of RA as a serious disease. In general, views on research participation, disease risk, and preventive intervention were similar between individuals at risk of RA or axSpA, suggesting generalizability of our findings in different at-risk populations. Despite the high expressed willingness of at-risk individuals to change their lifestyle, most rheumatologists do not advise lifestyle changes due to lack of time or lack of evidence on the effects of lifestyle change on disease risk. In contrast, rheumatologists seem more willing to start preventive medication than at-risk individuals.

## Supplementary information

**Additional file 1.** Survey for RA-risk participants. Copy of the survey that was sent out to study participants at risk of rheumatoid arthritis.

**Additional file 2.** Survey for axSpA-risk participants. Copy of the survey that was sent out to study participants at risk of axial spondyloarthritis.

**Additional file 3.** Survey for health care professionals. Copy of the survey that was sent out to rheumatologists.

## Data Availability

The datasets used and/or analyzed during the current study are available from the corresponding author on reasonable request.

## Abbreviations

ACPA: Anti-citrullinated protein antibodies
AMC: Academic Medical Center Amsterdam
axSpA: Axial spondyloarthritis
CI: Confidence interval
FDRs: First-degree relatives
GEE: Generalized estimating equations
IQR: Interquartile range
OR: Odds ratio
RA: Rheumatoid arthritis
RF: Rheumatoid factor
SD: Standard deviation
SpA: Spondyloarthritis
VAS: Visual analog scale

## References

[1] Gerlag DM, Norris JM, Tak PP (2016). Towards prevention of autoantibody-positive rheumatoid arthritis: from lifestyle modification to preventive treatment. Rheumatology (Oxford).

[2] Gerlag DM, Raza K, van Baarsen LG, Brouwer E, Buckley CD, Burmester GR (2012). EULAR recommendations for terminology and research in individuals at risk of rheumatoid arthritis: report from the Study Group for Risk Factors for Rheumatoid Arthritis. Ann Rheum Dis.

[3] van Boheemen L, van Schaardenburg D (2019). Predicting rheumatoid arthritis in at-risk individuals. Clin Ther.

[4] Cope AP (2019). Considerations for optimal trial design for rheumatoid arthritis prevention studies. Clin Ther.

[5] Kaipiainen-Seppanen O, Kautiainen H (2006). Declining trend in the incidence of rheumatoid factor-positive rheumatoid arthritis in Finland 1980-2000. J Rheumatol.

[6] Zaccardelli A, Friedlander HM, Ford JA, Sparks JA (2019). Potential of lifestyle changes for reducing the risk of developing rheumatoid arthritis: is an ounce of prevention worth a pound of cure?. Clin Ther.

[7] de Hair MJ, Landewe RB, van de Sande MG, van Schaardenburg D, van Baarsen LG, Gerlag DM (2013). Smoking and overweight determine the likelihood of developing rheumatoid arthritis. Ann Rheum Dis.

[8] Bos WH, Dijkmans BA, Boers M, van de Stadt RJ, van Schaardenburg D (2010). Effect of dexamethasone on autoantibody levels and arthritis development in patients with arthralgia: a randomised trial. Ann Rheum Dis.

[9] Gerlag DM, Safy M, Maijer KI, Tang MW, Tas SW, Starmans-Kool MJF (2019). Effects of B-cell directed therapy on the preclinical stage of rheumatoid arthritis: the PRAIRI study. Ann Rheum Dis.

[10] Al-Laith M, Jasenecova M, Abraham S, Bosworth A, Bruce IN, Buckley CD (2019). Arthritis prevention in the pre-clinical phase of RA with abatacept (the APIPPRA study): a multi-centre, randomised, double-blind, parallel-group, placebo-controlled clinical trial protocol. Trials..

[11] Naderi SH, Bestwick JP, Wald DS (2012). Adherence to drugs that prevent cardiovascular disease: meta-analysis on 376,162 patients. Am J Med.

[12] Smith SG, Sestak I, Forster A, Partridge A, Side L, Wolf MS (2016). Factors affecting uptake and adherence to breast cancer chemoprevention: a systematic review and meta-analysis. Ann Oncol.

[13] Finckh A, Escher M, Liang MH, Bansback N (2016). Preventive treatments for rheumatoid arthritis: issues regarding patient preferences. Curr Rheumatol Rep.

[14] Novotny F, Haeny S, Hudelson P, Escher M, Finckh A (2013). Primary prevention of rheumatoid arthritis: a qualitative study in a high-risk population. Joint Bone Spine.

[15] Simons G, Stack RJ, Stoffer-Marx M, Englbrecht M, Mosor E, Buckley CD (2018). Perceptions of first-degree relatives of patients with rheumatoid arthritis about lifestyle modifications and pharmacological interventions to reduce the risk of rheumatoid arthritis development: a qualitative interview study. BMC Rheumatol..

[16] Harrison M, Spooner L, Bansback N, Milbers K, Koehn C, Shojania K (2019). Preventing rheumatoid arthritis: preferences for and predicted uptake of preventive treatments among high risk individuals. PLoS One.

[17] Munro S, Spooner L, Milbers K, Hudson M, Koehn C, Harrison M (2018). Perspectives of patients, first-degree relatives and rheumatologists on preventive treatments for rheumatoid arthritis: a qualitative analysis. BMC Rheumatol.

[18] Mosor E, Stoffer-Marx M, Steiner G, Raza K, Stack RJ, Simons G, et al. I Would Never Take Preventive Medication! Perspectives and Information Needs of People Who Underwent Predictive Tests for Rheumatoid Arthritis. Arthritis Care Res (Hoboken). 2020;72(3):360–8. 10.1002/acr.23841. 10.1002/acr.23841 PMC7064954 30710453

[19] Brown MA, Laval SH, Brophy S, Calin A (2000). Recurrence risk modelling of the genetic susceptibility to ankylosing spondylitis. Ann Rheum Dis.

[20] Turina MC, de Winter JJ, Paramarta JE, Gamala M, Yeremenko N, Nabibux MN (2016). Clinical and imaging signs of spondyloarthritis in first-degree relatives of HLA-B27-positive ankylosing spondylitis patients: the pre-spondyloarthritis (pre-SpA) cohort study. Arthritis Rheumatol.

[21] de Winter JJ, de Jong HM, Nieuwkerk PT, van der Horst-Bruinsma IE, Baeten DL, van de Sande MG (2019). First-degree relatives of axial spondyloarthritis patients of the pre-SpA cohort would consider using medication in a preventive setting. Clin Rheumatol.

[22] Bos WH, Wolbink GJ, Boers M, Tijhuis GJ, de Vries N, van der Horst-Bruinsma IE (2010). Arthritis development in patients with arthralgia is strongly associated with anti-citrullinated protein antibody status: a prospective cohort study. Ann Rheum Dis.

[23] van de Stadt LA, Witte BI, Bos WH, van Schaardenburg D (2013). A prediction rule for the development of arthritis in seropositive arthralgia patients. Ann Rheum Dis.

[24] van Beers-Tas MH, Ter Wee MM, van Tuyl LH, Maat B, Hoogland W, Hensvold AH (2018). Initial validation and results of the Symptoms in Persons At Risk of Rheumatoid Arthritis (SPARRA) questionnaire: a EULAR project. RMD Open.

[25] Sepriano A, Ramiro S, van der Heijde D, van Gaalen F, Hoonhout P, Molto A (2020). What is axial spondyloarthritis? A latent class and transition analysis in the SPACE and DESIR cohorts. Ann Rheum Dis.

[26] Rantapaa Dahlqvist S, Andrade F (2019). Individuals at risk of seropositive rheumatoid arthritis: the evolving story. J Intern Med.

