# Supplementary material for: Patients’ and rheumatologists’ perceptions on preventive intervention in rheumatoid arthritis and axial spondyloarthritis
Source: Arthritis Res Ther. 2020 Sep 15;22:217. doi: 10.1186/s13075-020-02314-9 (PMC7493385; doi:10.1186/s13075-020-02314-9)
Supplement: Supplementary file 1 — Additional file 1. Survey for RA-risk participants. Copy of the survey that was sent out to study participants at risk of rheumatoid arthritis. [file 13075_2020_2314_MOESM1_ESM.docx]

[
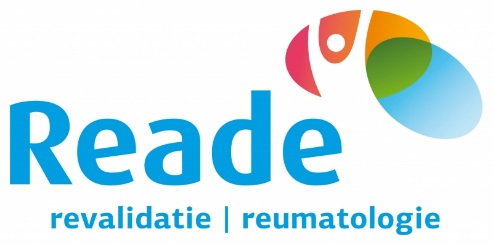
](https://www.google.nl/url?sa=i&rct=j&q=&esrc=s&source=images&cd=&cad=rja&uact=8&ved=2ahUKEwiH5ovNueHaAhWMEVAKHdSNDqoQjRx6BAgBEAU&url=https://www.revalidatie.nl/revalideren/member-r/reade-overtoom&psig=AOvVaw2yl6LNUHc8dFtJjmZ3Pb5V&ust=1525158845581385)

**Would you like to fill out a questionnaire?**

You are participating in medical research on persons who have an increased risk of developing rheumatoid arthritis, or RA. RA is a chronic inflammatory disease that affects the joints, which is accompanied by pain, stiffness and reduced functioning.

There is no **preventive treatment** for RA available yet. Preventive treatment is medication that can prevent you from developing RA. We would like to know if you would be willing to use any preventive medication.

Lifestyle affects the risk of developing RA, although for most parts it is not well known to what extent. We would also like to know if you would be willing to make lifestyle changes.

We also ask about your views on participating in medical research. That is what this questionnaire is about.

**How does the questionnaire work?**

You will read a number of six examples in which to make a choice whether to take preventive medication were you in the situation described. Please indicate after each example whether you would use this medication or not.

These 6 examples are followed by 12 questions regarding the disease and participation in a medical scientific study. Finally, there are 14 questions about the willingness to make lifestyle changes.

Good to know

- For the entire questionnaire the following applies:
- All examples are hypothetical. Try to consider each example carefully.
- The duration that you would use the preventive medication is indefinitely in each example. This means that the medication only works as long as you use it.
- You can always stop with the preventive medication. Any side effects would stop as well.
- The preventive medication does not cost you any money.
- All answers are correct. Because this questionnaire is about your opinion.
- The questionnaire is completely anonymous.

Filling out this questionnaire will cost you approximately 20 minutes.

Thank you for your time.

Please start with example 1 on the next page.

**EXAMPLE 1**

**Example**

If you do not use preventive medication

You will have a 30% chance of developing RA. This means that 3 out of 10 individuals will develop RA.

If you do use preventive medication

- You will not develop RA
- The medication does not have any side effects

**Question 1:**

Would you use this preventive medication?

1. No
2. Probably not
3. I do not know
4. Probably yes
5. Yes

Did you circle an answer?

Please continue to example 2.

**EXAMPLE 2**

**Example**

If you do not use preventive medication

You will have a 70% chance of developing RA. This means that 7 out of 10 individuals will develop RA.

If you do use preventive medication

- You will not develop RA
- The medication does not have any side effects

**Question 2:**

Would you use this preventive medication?

1. No
2. Probably not
3. I do not know
4. Probably yes
5. Yes

Did you circle an answer?

Please continue to example 3.

**EXAMPLE 3**

**Example**

If you do not use preventive medication

You will have a 70% chance of developing RA. This means that 7 out of 10 individuals will develop RA.

If you do use preventive medication

- You will not develop RA
- The medication can possibly cause mild side effects. This side effects are not serious, but can be unpleasant. Like headache, itch, nausea or a stomach ache. If you stop with the preventive medication the side effects will stop as well

**Question 3:**

Would you use this preventive medication?

1. No
2. Probably not
3. I do not know
4. Probably yes
5. Yes

Did you circle an answer?

Please continue to example 4.

**EXAMPLE 4**

**Example**

If you do not use preventive medication

You will have a 30% chance of developing RA. This means that 3 out of 10 individuals will develop RA.

If you do use preventive medication

- You will not develop RA
- You will possibly get an infection with a virus, bacteria or fungus more easily than without the medication. Such an infection is usually not serious and will pass on its own. But sometimes you will need to use medication to treat the infection.

**Question 4:**

Would you use this preventive medication?

1. No
2. Probably not
3. I do not know
4. Probably yes
5. Yes

Did you circle an answer?

Please continue to example 5.

**EXAMPLE 5**

**Example**

If you do not use preventive medication

You will have a 70% chance of developing RA. This means that 7 out of 10 individuals will develop RA.

If you do use preventive medication

- You will not develop RA
- You will possibly get an infection with a virus, bacteria or fungus more easily than without the medication. Such an infection is usually not serious and will pass on its own. But sometimes you will need to use medication to treat the infection.

**Question 5:**

Would you use this preventive medication?

1. No
2. Probably not
3. I do not know
4. Probably yes
5. Yes

Did you circle an answer?

Please continue to example 6.

**EXAMPLE 6**

**Example**

If you do not use preventive medication

You will have a 70% chance of developing RA. This means that 7 out of 10 individuals will develop RA.

If you do use preventive medication

- You will still have a 70% chance of developing RA, but if you develop it, it will start 10 years later;
- The medication does not have any side effects

**Question 6:**

Would you use this preventive medication?

1. No
2. Probably not
3. I do not know
4. Probably yes
5. Yes

Did you circle an answer?

Please continue to the question below.

**Question 7:**

If you consider either using preventive medication or not, what is the most important factor for you. Please circle only 1 answer.

1. The costs must be low
2. The medication must not have side effects
3. It must be certain that the medication can prevent RA
4. The medication must be easy to swallow
5. It must be very likely that I will develop RA
6. Other, namely ..

Did you circle an answer?

Please continue to the statements below.

**AGREE OR DISAGREE?**

You have an increased risk of developing RA. We would like to understand what effect this knowledge has on you. The following questions concern this.

The questions consist of statements. You can indicate on a scale of 0 to 10 whether you agree with the statement. To do so, tick a box between 0 (totally disagree) and 10 (totally agree).

**Question 8**:
The thought of developing RA preoccupies me.

0 1 2 3 4 5 6 7 8 9 10

Totally disagree Totally agree

**Question 9**:
I am certain that I will develop RA.

0 1 2 3 4 5 6 7 8 9 10

Totally disagree Totally agree

**Question 10**:
RA is a severe disease.

0 1 2 3 4 5 6 7 8 9 10

Totally disagree Totally agree

**Question 11**:
I am worried that I have an increased risk of developing RA

0 1 2 3 4 5 6 7 8 9 10

Totally disagree Totally agree

**Question 12**:

By participating in this research, I feel that I am extra confronted with the fact that I have an increased risk to develop RA.

0 1 2 3 4 5 6 7 8 9 10

Totally disagree Totally agree

If you gave a score of 1 or higher on the previous question, please fill in the following question.

**Question 13**:
How objectionable is it for you to be extra confronted with the risk to develop RA by participation in this cohort?

0 1 2 3 4 5 6 7 8 9 10

Not objectionable Very objectionable

**Question 14**:
By participating in this research there is more attention for my complaints.

0 1 2 3 4 5 6 7 8 9 10

Totally disagree Totally agree

**Question 15**:
I think that by participating in this study I will receive earlier and better medical care upon RA development than if I did not participate in this study.

0 1 2 3 4 5 6 7 8 9 10

Totally disagree Totally agree

**Question 16**:
What was the reason for you to participate in this research?

**Question 17**:
What is it like for you to know that you have an increased risk of developing RA?

**Question 18**:
How do you experience taking part in research into the development of RA?

**Lifestyle questions**

The following questions are about the willingness to make lifestyle changes. Lifestyle affects the risk of developing RA, but for most parts it is not well known to what extent. In the following questions, it is assumed that the risk of developing RA is halved, whereas in reality this effect could be greater or smaller.

Suppose you have a 50% chance of developing RA. This means that 5 out of 10 people develop RA. And suppose by making some changes to your current lifestyle you can halve that chance, and have a 25% chance of developing RA.

The following questions are about your willingness to make lifestyle changes to reduce your risk. So keep in mind with every question that you reduce your risk of developing RA by 50%.

**1.** Do you smoke? **yes / no**

**1a.** If you smoke, would you be willing to stop? **yes / no**

**1b.** Please indicate how willing you are to stop

0 1 2 3 4 5 6 7 8 9 10

Not willing at all Very willing

**2.** Are you currently doing moderate exercise 5 days per week for 30 minutes (for example walking or biking)? **yes / no**

**2a.** Are you currently doing intensive exercise 2 days per week for 20 minutes (sports, muscle strengthening activities)? **yes / no**

**2b**. Suppose you answered no to any of the above questions, would you be willing to do moderate exercise 5 days per week for 30 minutes AND intensive exercise 2 days per week for 20 minutes? **yes / no**

**2c**. Please indicate how willing you are to exercise moderately for 30 minutes 5 days a week AND intensively for 20 minutes twice a week

0 1 2 3 4 5 6 7 8 9 10

Not willing at all Very willing

**3.** Do you eat meat and chicken? **yes / no**
If you answered no, continue to question 4.

**3a.** Please indicate how willing you are to stop eating meat and chicken at all

0 1 2 3 4 5 6 7 8 9 10

Not willing at all Very willing

**4.** Do you eat fish? **yes / no**

If you answered no, continue to question 5.

**4a.** Please indicate how willing you are to stop eating fish at all

0 1 2 3 4 5 6 7 8 9 10

Not willing at all Very willing

**5.** Do you eat dairy, eggs and cheese? **yes / no**
If you answered no, continue to question 6.

**5a.** Please indicate how willing you are to stop eating dairy, eggs and cheese at all

0 1 2 3 4 5 6 7 8 9 10

Not willing at all Very willing

**6.** Do you eat added sugars (such as sweets like candy, biscuits, etc)? **yes / no**

If you answered no, continue to question 7.

**6a.** Please indicate how willing you are to stop eating added sugars at all

0 1 2 3 4 5 6 7 8 9 10

Not willing at all Very willing

**7.** Do you always choose the whole grain option of products (such as whole grain bread, pasta and brown rice)? **yes / no**

If you answered yes, continue to question 8.

**7a.** Please indicate how willing you are to eat only whole grain products

0 1 2 3 4 5 6 7 8 9 10

Not willing at all Very willing

**8.** Do you currently eat 250 grams of vegetables or more per day? **yes / no**

If you answered yes, continue to question 9.

**8a.** Please indicate how willing you are to eat at least 250 grams of vegetables per day

0 1 2 3 4 5 6 7 8 9 10

Not willing at all Very willing

**9.** Do you currently eat 2 pieces of fruit or more per day? **yes / no**

If you answered yes, continue to question 10.

**9.a** Please indicate how willing you are to eat at least 2 pieces of fruit per day

0 1 2 3 4 5 6 7 8 9 10

Not willing at all Very willing

**10.** Do you currently eat legumes at least once per week (such as chickpeas, lentils, white beans, brown beans, etc.)? **yes / no**

If you answered yes, continue to question 11.

**10a**. Please indicate how willing you are to eat legumes at least once per week

0 1 2 3 4 5 6 7 8 9 10

Not willing at all Very willing

**11.** Do you drink alcohol? **yes / no**

**11a.** Do you ever drink more than 1 glass of alcohol per day? **yes / no**

If you answered no, continue to question 12.

**11c.** Please indicate how willing you are to limit your alcohol consumption to a maximum of 1 glass per day

0 1 2 3 4 5 6 7 8 9 10

Not willing at all Very willing

**12.** Do you drink sodas and fruit juices? **yes / no**

If you answered no, continue to question 13.

**12a.** Please indicate how willing you are to stop drinking sodas and fruit juices at all

0 1 2 3 4 5 6 7 8 9 10

Not willing at all Very willing

**13.** Do you only drink water and tea (without sweeteners or sugar)? **yes / no**
If you answered yes, continue to question 14.

**13a.** Please indicate how willing you are to drink only water or tea without sweeteners or sugar

0 1 2 3 4 5 6 7 8 9 10

Not willing at all Very willing

**14.** Now that you've gone through all 13 lifestyle questions, how many of the lifestyle changes would you be willing to make in total?

1 2 3 4 5 6 7 8 9 10 11 12 13

**Thank you for your time!**
