# Supplementary material for: Patients’ and rheumatologists’ perceptions on preventive intervention in rheumatoid arthritis and axial spondyloarthritis
Source: Arthritis Res Ther. 2020 Sep 15;22:217. doi: 10.1186/s13075-020-02314-9 (PMC7493385; doi:10.1186/s13075-020-02314-9)
Supplement: Supplementary file 3 — Additional file 3. Survey for health care professionals. Copy of the survey that was sent out to rheumatologists. [file 13075_2020_2314_MOESM3_ESM.docx]

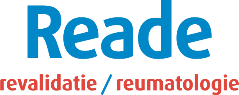


**Content**

At Reade we are currently conducting research into the willingness of patients with an increased risk of developing rheumatoid arthritis (RA) to take preventive medication or make lifestyle changes. In addition, we would like to investigate the rheumatologist's willingness to start preventive interventions.

**How the survey works**

We give 6 examples where you can choose whether you would prescribe preventive medication to your patient in that situation. This is followed by 5 questions about preventive intervention with medication or lifestyle advice.

**For the entire questionnaire the following applies:**

- All cases are fictional. Try to imagine that the case described concerns a patient of yours.
- Also imagine that the prescribed medication in the example is (cost-) effective, according to at least one randomized trial.
- The preventive medication must be used indefinitely (in the lowest possible dosage).
- For comparability, the questions have been formulated in the same way as for the patients.
- All answers are correct because this questionnaire is about your opinion

**Filling out this questionnaire will cost you approximately 20 minutes.**

Thank you for your time.

**EXAMPLE 1**

**Example**

If your patient does not use preventive medication

Your patients will have a 30% chance of developing RA within 3 years.

If your patient does use preventive medication

- Your patient will not develop RA
- The medication does not have any side effects

**Question 1:**

Would you start this preventive medication?

1. No
2. Probably not
3. I do not know
4. Probably yes
5. Yes

Explain your answer if desired: _____________________________________

**EXAMPLE 2**

**Example**

If your patient does not use preventive medication

Your patient will have a 70% chance of developing RA within 3 years.

If your patient does use preventive medication

- Your patient will not develop RA
- The medication does not have any side effects

**Question 2:**

Would you start this preventive medication?

1. No
2. Probably not
3. I do not know
4. Probably yes
5. Yes

Explain your answer if desired: _____________________________________

**EXAMPLE 3**

**Example**

If your patient does not use preventive medication

Your patient will have a 70% chance of developing RA within 3 years.

If your patient does use preventive medication

- Your patient will not develop RA
- The medication can possibly cause mild side effects. This side effects are not serious, but can be unpleasant. Like headache, itch, nausea or a stomach ache. If you stop with the preventive medication the side effects will stop as well

**Question 3:**

Would you start this preventive medication?

1. No
2. Probably not
3. I do not know
4. Probably yes
5. Yes

Explain your answer if desired: _____________________________________

**EXAMPLE 4**

**Example**

If your patient does not use preventive medication

Your patient will have a 30% chance of developing RA within 3 years.

If your patient does use preventive medication

- Your patient will not develop RA
- Your patient will possibly get an infection with a virus, bacteria or fungus more easily than without the medication. Such an infection is usually not serious and will pass on its own. But sometimes your patient will need to use medication to treat the infection.

**Question 4:**

Would you start this preventive medication?

1. No
2. Probably not
3. I do not know
4. Probably yes
5. Yes

Explain your answer if desired: _____________________________________

**EXAMPLE 5**

**Example**

If your patient does not use preventive medication

Your patient will have a 70% chance of developing RA within 3 years.

If your patient does use preventive medication

- Your patient will not develop RA
- Your patient will possibly get an infection with a virus, bacteria or fungus more easily than without the medication. Such an infection is usually not serious and will pass on its own. But sometimes your patient will need to use medication to treat the infection.

**Question 5:**

Would you start this preventive medication?

1. No
2. Probably not
3. I do not know
4. Probably yes
5. Yes

Explain your answer if desired: _____________________________________

**EXAMPLE 6**

**Example**

If your patient does not use preventive medication

Your patient will have a 70% chance of developing RA within 3 years.

If your patient does use preventive medication

- Your patient will still have a 70% chance of developing RA, but if your patient develops it, it will start 10 years later;
- The medication does not have any side effects

**Question 6:**

Would you start this preventive medication?

1. No
2. Probably not
3. I do not know
4. Probably yes
5. Yes

Explain your answer if desired: _____________________________________

**Question 7**
How high must your patient's risk of RA development within 3 years be before you would start preventive medication?

10% 20% 30% 40% 50% 60% 70% 80% 90% 100%

I would never start preventive medication.

Explain your answer if desired: _____________________________________

**Question 8:**
If you consider either starting preventive medication or not, what is the most important factor for you. Please circle only 1 answer.

1. The costs must be low
2. The medication must not have side effects
3. It must be certain that the medication can prevent RA
4. The medication must be easy to swallow
5. It must be very likely that your patient will develop RA
6. Other, namely

Explain your answer if desired: _____________________________________

**Lifestyle questions**

The final questions are about lifestyle advice. Lifestyle affects the risk of developing RA, but for most parts it is not well known to what extent.

**Question 1a**

To what percentage of your patients with an increased risk of RA (such as seropositive arthralgia or clinically suspect arthralgia) do you give lifestyle advice with the aim of lowering the risk of RA?

<10%

10-30%

30-50%

50-70%

>70%

Explain your answer if desired: _____________________________________

**Question 1b**

If you ever give lifestyle advice, which one(s)? Multiple answers can be ticked:

stop smoking

exercise more

eat less meat

eat less fish

consume less dairy

eat less added sugars

eat more fibers (whole grain products)

eat more vegetables (at least 250 grams per day)

eat more fruit (at least 2 pieces per day)

eat more legumes (at least once per week)

drink less alcohol (maximum 1 glass per day)

drink less sodas

drink only water or tea

other, namely:

Explain your answer if desired: _____________________________________

**Vraag 1c**

If you do not give lifestyle advice, why not? Multiple answers can be ticked:

I do not have enough knowledge about lifestyle to give lifestyle advice

I do not believe in it, there is too little evidence for lifestyle advice

I do not have enough time to give lifestyle advice

It is not my job to give lifestyle advice

Other, namely:

Explain your answer if desired: _____________________________________

**Finally, please fill in your age and sex.**

Age: __ years

Sex: M / F

**Thank you for your time!**
